# Transient Hypermutagenesis Accelerates the Evolution of Legume Endosymbionts following Horizontal Gene Transfer

**DOI:** 10.1371/journal.pbio.1001942

**Published:** 2014-09-02

**Authors:** Philippe Remigi, Delphine Capela, Camille Clerissi, Léna Tasse, Rachel Torchet, Olivier Bouchez, Jacques Batut, Stéphane Cruveiller, Eduardo P. C. Rocha, Catherine Masson-Boivin

**Affiliations:** 1 INRA, Laboratoire des Interactions Plantes-Microorganismes (LIPM), UMR441, Castanet-Tolosan, France; 2 CNRS, Laboratoire des Interactions Plantes-Microorganismes (LIPM), UMR2594, Castanet-Tolosan, France; 3 CNRS-UMR 8030 and Commissariat à l'Energie Atomique CEA/DSV/IG/Genoscope LABGeM, Evry, France; 4 INRA, UMR1388 Génétique, Physiologie et Systèmes d'Elevage, Castanet-Tolosan, France; 5 GeT-PlaGe, Genotoul, INRA Auzeville, Castanet-Tolosan, France; 6 CNRS UMR3525, Paris, France; 7 Microbial Evolutionary Genomics, Institut Pasteur, Paris, France; The University of North Carolina at Chapel Hill, United States of America

## Abstract

Horizontal gene transfer (HGT) is an important mode of adaptation and diversification of prokaryotes and eukaryotes and a major event underlying the emergence of bacterial pathogens and mutualists. Yet it remains unclear how complex phenotypic traits such as the ability to fix nitrogen with legumes have successfully spread over large phylogenetic distances. Here we show, using experimental evolution coupled with whole genome sequencing, that co-transfer of *imuABC* error-prone DNA polymerase genes with key symbiotic genes accelerates the evolution of a soil bacterium into a legume symbiont. Following introduction of the symbiotic plasmid of *Cupriavidus taiwanensis*, the *Mimosa* symbiont, into pathogenic *Ralstonia solanacearum* we challenged transconjugants to become *Mimosa* symbionts through serial plant-bacteria co-cultures. We demonstrate that a mutagenesis *imuABC* cassette encoded on the *C. taiwanensis* symbiotic plasmid triggered a transient hypermutability stage in *R. solanacearum* transconjugants that occurred before the cells entered the plant. The generated burst in genetic diversity accelerated symbiotic adaptation of the recipient genome under plant selection pressure, presumably by improving the exploration of the fitness landscape. Finally, we show that plasmid *imuABC* cassettes are over-represented in rhizobial lineages harboring symbiotic plasmids. Our findings shed light on a mechanism that may have facilitated the dissemination of symbiotic competency among α- and β-proteobacteria *in natura* and provide evidence for the positive role of environment-induced mutagenesis in the acquisition of a complex lifestyle trait. We speculate that co-transfer of complex phenotypic traits with mutagenesis determinants might frequently enhance the ecological success of HGT.

## Abstract 2

Stress-responsive error-prone DNA polymerase genes transferred along with key symbiotic genes ease the evolution of a soil bacterium into a legume endosymbiont by accelerating adaptation of the recipient bacterial genome to its new plant host.

## Author Summary

Horizontal gene transfer has an extraordinary impact on microbe evolution and diversification, by allowing exploration of new niches such as higher organisms. This is the case for rhizobia, a group of phylogenetically diverse bacteria that form a nitrogen-fixing symbiotic relationship with most leguminous plants. While these arose through horizontal transfer of symbiotic plasmids, this in itself is usually unproductive, and full expression of the acquired traits needs subsequent remodeling of the genome to ensure the ecological success of the transfer. Here we uncover a mechanism that accelerates the evolution of a soil bacterium into a legume symbiont. We show that key symbiotic genes are co-transferred with genes encoding stress-responsive error-prone DNA polymerases that transiently elevate the mutation rate in the recipient genome. This burst in genetic diversity accelerates the symbiotic evolution process under selection pressure from the host plant. A more widespread involvement of plasmid mutagenesis cassettes in rhizobium evolution is supported by their overrepresentation in rhizobia-containing lineages. Our findings provide evidence for the role of environment-induced mutagenesis in the acquisition of a complex lifestyle trait and predict that co-transfer of complex phenotypic traits with mutagenesis determinants might help successful horizontal gene transfer.

## Introduction

Horizontal gene transfer (HGT) drives bacterial ecological diversification by providing genomes with new genes and functions [1]–[4]. Key changes in lifestyle can result from the acquisition by HGT of genes facilitating symbiosis, either mutualistic or parasitic, with eukaryotes [5],[6]. For example, the high virulence of *Shigella flexneri* and *Vibrio cholerae* results directly from the acquisition of virulence factors in mobile genetic elements [7],[8]. Mobile elements also confer traits that are involved in the establishment of mutualistic associations [9]. Many mobile genetic elements have narrow-host ranges and this favors horizontal transfer between closely related bacteria [10]. Transfer is also more likely to be successful when it concerns simple traits and takes place between closely related bacteria because this increases the probability of gene expression and integration in the host genetic background [11],[12]. Nevertheless, genetic transfer of complex traits between very distantly related taxa has an important role in bacterial evolution [13]. Transfer of complex traits is expected to lead to bursts of adaptation in the newly acquired traits. For example, acquisition of type 3 secretion systems by plant-associated pathogenic bacteria was followed by the replacement of the needle by pilus-like structures more adequate to interact with the plant cell wall [14]. Yet the environmental and genetic factors that determine the evolutionary success of HGT remain poorly understood.

Soil bacteria termed rhizobia are remarkable examples of bacteria that arose through HGT. Rhizobia are phylogenetically dispersed bacteria currently distributed in at least 13 saprophyte- and pathogen-containing genera of α- and β-proteobacteria [15], which have evolved the environmentally essential function of fixing atmospheric nitrogen in symbiosis with legumes. Rhizobial mutualistic symbiosis with legumes is a complex process involving three main steps: nodule organogenesis, intracellular infection, and nitrogen fixation [15],[16]. This endosymbiosis is controlled by a large number of genes in both partners including a set of essential nodulation and nitrogen fixation bacterial genes clustered in mobile genetic elements such as symbiotic plasmids or genomic islands [16],[17]. Horizontal transfer of essential symbiotic genes has been key in the conversion of soil bacteria into mutualistic symbionts of legumes [18]. Although compared phylogenies of rhizobia and nodulation genes predict that symbiotic genes have been transferred over large phylogenetic distances [19]–[21], transfer alone in lab conditions is usually unproductive between evolutionary distant taxa [22]–[24].

**Figure 1:**
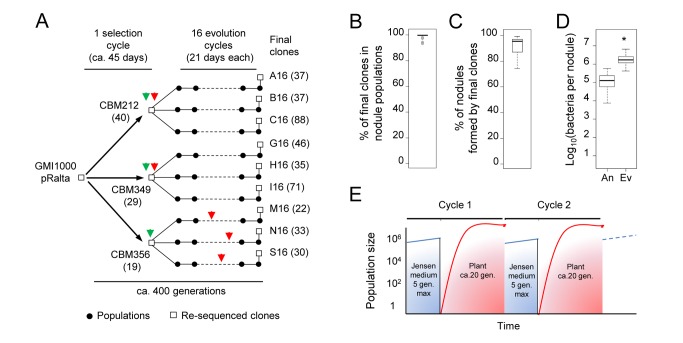
Experimental evolution of *R. solanacearum* into *Mimosa* symbionts. (A) *R. solanacearum* GMI1000 containing the *C. taiwanensis* symbiotic pRalta plasmid was evolved under *M. pudica* selection pressure. In a first step, three spontaneous *M. pudica*-nodulating derivatives of *GMI1000* pRalta, CBM212, CBM349, and CBM356 (selection cycle), were selected using *M. pudica* as a trap [24]. Nine independent lineages have been then derived from CBM212 (A–C), CBM349 (G–I), and CBM356 (M, N, S) using serial *M. pudica*-bacteria co-culture cycles of 21 days (evolution cycles) [24],[25]. Green and red arrow heads indicate activation of nodulation and intracellular infection, respectively [24],[25]. Numbers between brackets indicate the total number of point mutations detected in each clone relative to its closest re-sequenced ancestor. Point mutations are available on the Microscope platform (https://www.genoscope.cns.fr/agc/microscope/expdata/evoProject.php). (B–D) Nodulation and infection have been dramatically improved over 16 evolution cycles. *In planta* fitness (B) and nodulation competitiveness (C) of final clones relative to their respective nodulating ancestors, following equal co-inoculation of each of the nine final/ancestral pairs. Nodule infectiveness (D) of final clones (Ev) as compared to ancestors (An). Graphs summarize data from [25],[26]. *p-value (t-test) <0.05. (E) In each cycle, bacteria were inoculated in the Jensen plant medium. Root nodules, which appeared from 5 days after inoculation, were each induced by a single bacterial cell that subsequently multiplied within nodule tissue [56]. In the selection and evolution cycles bacteria spent ∼21 days and from a few days up to 14 days in the plant medium, respectively. Population sizes are estimates. gen., generations. Raw data are provided in Data S1.

To get an insight into the evolutionary mechanisms that facilitated rhizobium diversification, we experimentally replayed the evolution of rhizobia. We introduced the symbiotic conjugative plasmid pRalta (0.5 Mb) of the *Mimosa* symbiont *Cupriavidus taiwanensis* into the plant pathogen *Ralstonia solanacearum* GMI1000 and used *Mimosa* to trap symbiotic variants of this still pathogenic and non nodulating ancestor [24]. Spontaneous nodulating variants of the chimeric *Ralstonia* GMI1000pRalta were then submitted to serial and parallel *ex planta-in planta* (*Mimosa*) passages (see Figure 1A). This alternation recapitulated the shifts between free-living and symbiotic lives that have shaped the natural evolution of rhizobia. Evolution was surprisingly fast since the first two major symbiotic steps, nodulation and intracellular infection, were not only activated but also dramatically improved over 17 cycles (∼400 generations) in all lineages (see Figure 1A–1D) [24]–[26]. A first level of nodulation and infection capacity was reached via inactivation of regulatory or structural genes of the virulence pathway of the recipient genome, revealing that acquisition of symbiotic proficiency in phylogenetically distant bacterial lineages requires further recipient genome remodeling [24],[25].

Here we provide evidence for a mechanism facilitating the dissemination of rhizobial symbiotic competency over large phylogenetic distances *in natura*. The symbiotic plasmid of *C. taiwanensis* bears stress-responsive error-prone *imuABC* DNA polymerase genes that, following transfer to *R. solanacearum*, accelerate the adaptation of the recipient genome to its new plant host. The phylogenetic distribution of *imuABC* cassettes supports their role in the evolution of rhizobia that arose via plasmid transfer.

## Results and Discussion

### Chimeric *Ralstonia* Underwent Environment-Induced Hypermutagenesis during Their Experimental Evolution into Legume Symbionts

Whole genome re-sequencing of the nine final clones of the evolution experiment revealed between 41 to 128 point mutations scattered in each genome and absent from the chimeric GMI1000pRalta ancestor (Figure 1A). This large number of mutations prompted us to test the hypothesis that mutators had arisen during our experiment. Yet, no mutation was detected in the DNA repair system of adapted clones. Furthermore fluctuation tests [27], which allow measuring the mutation rate of growing bacteria, confirmed that neither the chimeric ancestor GMI1000pRalta nor the three first nodulating clones CBM212, CBM349, and CBM356 (Figure 1A) were constitutive hypermutators (Figure S1). These results suggest that transient hypermutagenesis generated genetic diversity during the evolution experiment.

**Figure 2:**
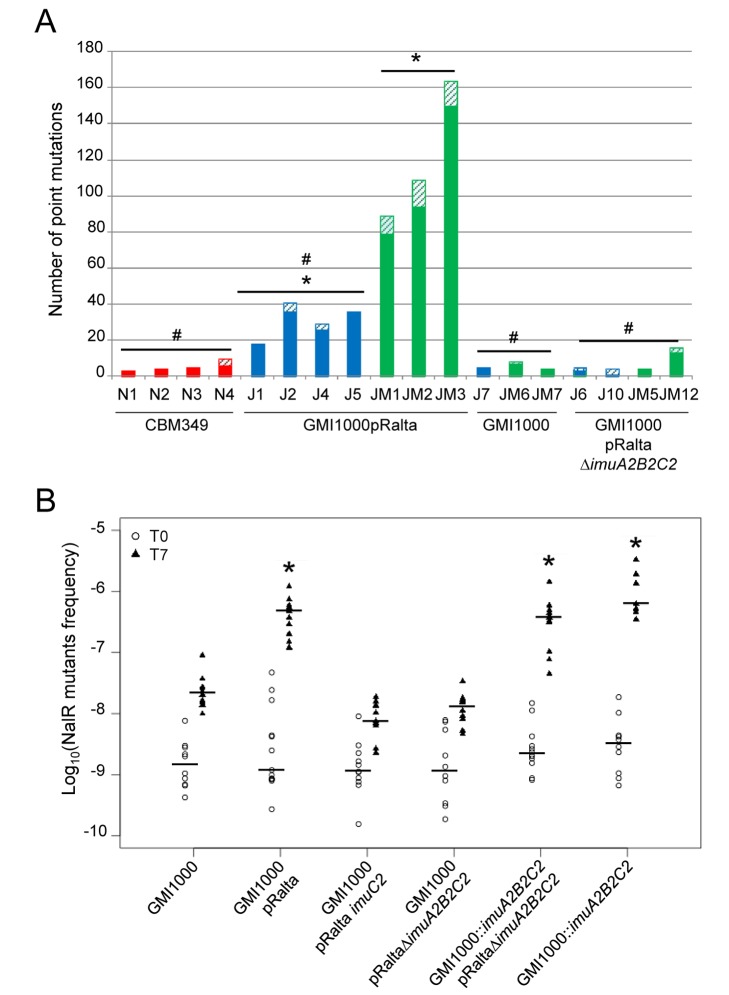
Environment-induced and pRalta-dependent hypermutability of chimeric *Ralstonia*. (A) Number of point mutations detected in pools of 19 or 20 bacteria isolated from nodules (red bars), Jensen medium (blue bars), or Jensen medium plus *Mimosa* (green bars). Full and hatched bars represent mutations present in one or two clones and in more than two clones, respectively. * and # Indicate values significantly different from N1–N4 values (J1–J5: Wilcoxon test *p* = 0.02, ANOVA *p*<0.005; JM1–JM3: Wilcoxon test *p*<0.05; ANOVA *p*<0.002); and from JM1–JM3 (J1–J5: Wilcoxon test *p*<0.05; ANOVA *p*<0.005; N1–N4: Wilcoxon test *p*<0.05; ANOVA *p*<0.002; J7–JM7:Wilcoxon test *p*<0.05; ANOVA *p*<0.005; J6–JM12:Wilcoxon test *p*<0.05; ANOVA *p*<0.005), respectively. Details are provided in Tables S1 and S4. (B) Frequency of spontaneous NalR mutants in bacterial populations of GMI1000, GMI1000pRalta, *imuA2B2C2* mutants (lanes 3 and 4), or complemented *imuA2B2C2* mutants (lanes 5 and 6) in Jensen medium. Bacterial strains were grown overnight in rich medium and then transferred into Jensen medium. The frequency of NalR mutants was measured before (T0) and after (T7) a 7-day incubation. Each dot represents an independent measurement. Horizontal bars represent medians. *Indicates T7 values significantly different from GMI1000 (two-tailed Kruskal-Wallis test, *p*<0.05). T0 values are not significantly different. Raw data are provided in Data S2.

In each cycle plants in tubes were inoculated with bacteria, which diffused in the carbon-free and nitrogen-free plant culture Jensen medium before entering the root and multiplying within the newly induced nodules (Figure 1E). To determine whether genetic diversification occurred outside or inside the plant, we replayed a single evolution cycle several times independently and re-sequenced pools of 19 or 20 clones randomly isolated from the culture medium or from nodules 21 days after inoculation (single evolution cycle and re-sequencing [SEC&R] assay). Three to ten different point mutations were found in pools isolated from nodules following inoculation by the nodulating ancestor CBM349 (Figure 2A; Table S1). Using the number of observed synonymous mutations, the genome sequencing coverage and the density of synonymous sites, we estimated the *in planta* synonymous point-mutation rate to 0.6–2×10^−11^ per bp per generation, slightly lower than estimations from long-term *Escherichia coli* evolution experiments (4–14×10^−11^ per bp per generation) [28]. By contrast, the non-nodulating ancestor GMI1000pRalta accumulated in the same period of time on average five and 20 times more mutations when incubated in the medium alone or in the medium with *Mimosa* seedlings, respectively (Figure 2A; Table S1). In bacterial populations, both strong selective pressures and hypermutability can trigger fast fixation of mutations [29]. We found no evidence for widespread positive selection in detected mutations and very few convergent mutations either in our SEC&R assay or in the final evolved clones. Instead, many synonymous mutations and an excess of these over non-synonymous mutations indicated an imprint of purifying selection (Table S2). This finding suggests that most detected mutations are not adaptive and have achieved fixation by hitchhiking with adaptive ones. Altogether these results showed that bacteria were subjected to hypermutagenesis, presumably stress-induced, *ex planta* but probably not in nodules. Further work is needed to identify the inducing environmental factors. We speculate that nutrient starvation, a condition frequently encountered in the soil [30], could be involved since it was endured by bacteria in both media. Bacteria showed very few divisions within 21 days (Figure S2C–S2E) and rapidly entered stationary then mortality phases (Figure S2A and S2B). The production by *Mimosa* plants of reactive oxygen species or other toxic compounds could account for the increased genetic diversity observed in the presence of the plant.

### Transferred ImuABC Error-Prone DNA Polymerases Trigger Environment-Induced Hypermutability

Few (5–7) mutations were found in pools of the *R. solanacearum* wild-type strain GMI1000 incubated in the medium with or without the plant, revealing the key role of pRalta in hypermutagenesis (Figure 2A; Table S1). The pRalta plasmid harbors a locus of three genes encoding a protein of unknown function (ImuA2), a Y-family DNA polymerase (ImuB2), and a C-family error-prone polymerase (ImuC2) [31]. This cassette, either complete or without the *imuA* gene, is widespread in bacteria and has been shown to mediate stress-induced mutagenesis as part of the SOS response [32]–[36]. The *imuA2B2C2* operon is preceded by a typical β/γ-proteobacterial LexA binding SOS box (CTGTN8ACAG) [34] in pRalta, and its expression depends on LexA, a negative regulator of the SOS response (Figure S3).

To investigate whether the *imuA2B2C2* cassette was responsible for hypermutability in GMI1000pRalta in our experimental conditions, we monitored the spontaneous appearance of nalidixic acid (NalR) resistant clones during incubation of the (non-growing) bacteria in Jensen medium. We checked that NalR mutants did not exhibit a growth advantage in Jensen medium by performing competition experiments between the chimeric strain GMI1000pRalta and spontaneous NalR derivatives. Competition experiments showed no increase in NalR mutant frequency because of selection (competitive index NalR/WT 0.99±0.32, *p*>0.5, one-sample t-test), indicating that this assay faithfully measured mutability. In line with whole-genome re-sequencing results, NalR mutant frequency in the medium was ∼15 times higher for GMI1000pRalta than for GMI1000 as soon as from three days of incubation (Figure S4A). Both deletion and insertion *imuA2B2C2* mutants of GMI1000pRalta exhibited a NalR mutant frequency comparable to that of GMI1000 seven days after incubation with (Figure S4B) or without (Figure 2B) the plant. The mutator phenotype was restored by complementing GMI1000 or GMI1000pRaltaΔ*imuA2B2C2* with the wild-type cassette under the control of its promoter (Figure 2B). A SEC&R assay on GMI1000pRaltaΔ*imuA2B2C2* confirmed that the *imuA2B2C2* cassette mediates hypermutagenesis (Figure 2A). Hence hypermutagenesis observed in the Jensen plant medium was strictly dependent upon the pRalta *imuA2B2C2* products.

We tested whether the cassette leads to hypermutagensis in *C. taiwanensis* the primary pRalta host. Under the same experimental conditions we found no evidence of pRalta-dependent hypermutagenesis in *C. taiwanensis* harboring the pRalta plasmid (Table S1). Hypermutagenesis generates a large load of deleterious mutations [37] that may be compensated by adaptive mutations in poorly adapted, but not in well-adapted, clones [38]. *C. taiwanensis* may thus have evolved strategies to silence plasmid *imuABC*-based mutagenesis. Altogether this suggests that *imuA2B2C2*-mediated hypermutagenesis specifically occurs in a recipient genome following plasmid transfer in our experimental conditions.

### *imuABC*-Mediated Hypermutagenesis Accelerates the Symbiotic Evolution of *R. solanacearum*

**Figure 3:**
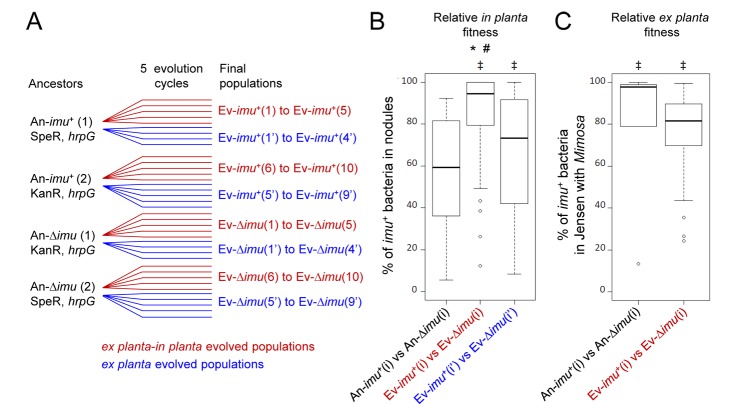
Evolvability of *imuA2B2C2* ^+^ populations. (A) Experimental evolution of *imuA2B2C2* ^+^ and Δ*imuA2B2C2* nodulating chimeric *Ralstonia*. Each ancestor was evolved using serial *M.pudica*-bacteria co-culture cycles, either *ex planta-in planta* cycles of 21 days (nodule bacteria serving as inoculum in each cycle, red lines) or *ex planta* cycles of 7 days (rhizospheric bacteria serving as inoculum in each cycle, blue lines). For *ex planta* lineages, 7-day cycles were chosen since the mean time bacteria spent *ex planta* in the 16 cycle evolution experiment (Figure 1) was estimated to be 7 days. Ancestors were antibiotic resistant derivatives of a *hrpG* mutant of GMI1000pRalta. Four to five independent lineages have been derived from each ancestor. SpeR, spectinomycin-resistant strain. KanR, kanamycin-resistant strain. (B) Relative *in planta* fitness of *imuA2B2C2* ^+^ versus Δ*imuA2B2C2* populations following co-inoculations of final populations derived from *ex planta-in planta* lineages (red legend, all Ev-*imu* ^+^(i) versus Ev-Δ*imu*(i) pairs) or from *ex planta* lineages (blue legend, all Ev-*imu* ^+^(i′) versus Ev-Δ*imu*(i′) pairs). Nodule bacteria were counted 21 days after inoculation. *Indicates significant differences between ancestral *imu* ^+^ clones and evolved *imu^+^* populations (t-test, *p*<0.05). #Indicates significant differences between *Ev-imu* ^+^(i) (evolved *ex planta-in planta*) and Ev-*imu^+^*(i′) (evolved *ex planta*) populations (t-test, *p*<0.05). ‡Indicates significant differences between Ev-*imu* ^+^ and Ev-Δ*imu* populations for each series of competition experiments (either Ev-*imu^+^*(i) versus Ev-Δ*imu*(i) or Ev-*imu^+^*(i′) versus Ev-Δ*imu*(i′), t-test, *p*<0.001). See Figure S5B and S5C and Data S3 for details. (C) Relative *ex planta* fitness of *imuA2B2C2* ^+^ versus Δ*imuA2B2C2* populations following co-inoculations of final populations derived from *ex planta-in planta* lineages. Bacteria recovered from the Jensen medium were counted 7 days after inoculation in Gibson tubes containing *M. pudica* plants. *imuA2B2C2* ^+^ ancestors better survived in Jensen-*Mimosa* than Δ*imuA2B2C2*, in accordance with results presented in Figure S2A. ‡Indicates significant differences between *imu* ^+^ and Δ*imu* populations for each series of competition experiments (either An-*imu* ^+^(i) versus An-Δ*imu*(i) or Ev-*imu^+^*(i) versus Ev-Δ*imu*(i), t-test, *p*<0.01). No significant difference was observed between ancestral *imu* ^+^ clones and evolved *imu* ^+^ populations. Raw data are provided in Data S3.

To directly evaluate whether *imuA2B2C2*-dependent mutagenesis accelerates the evolution of chimeric *Ralstonia* into *Mimosa* symbionts, we replayed evolution using either *imuA2B2C2* ^+^ or Δ*imuA2B2C2* chimeras as ancestors. To shorten the experiment we bypassed the selection stage (Figure 1A) by using a nodulating (*hrpG*) background. Inactivation of this single regulatory gene allows elementary nodulation and infection in GMI1000pRalta [24]. We used two different regimes of selection, one involving serial *ex planta-in planta* passages and the other only *ex planta* passages (Figure 3A).

**Figure 4:**
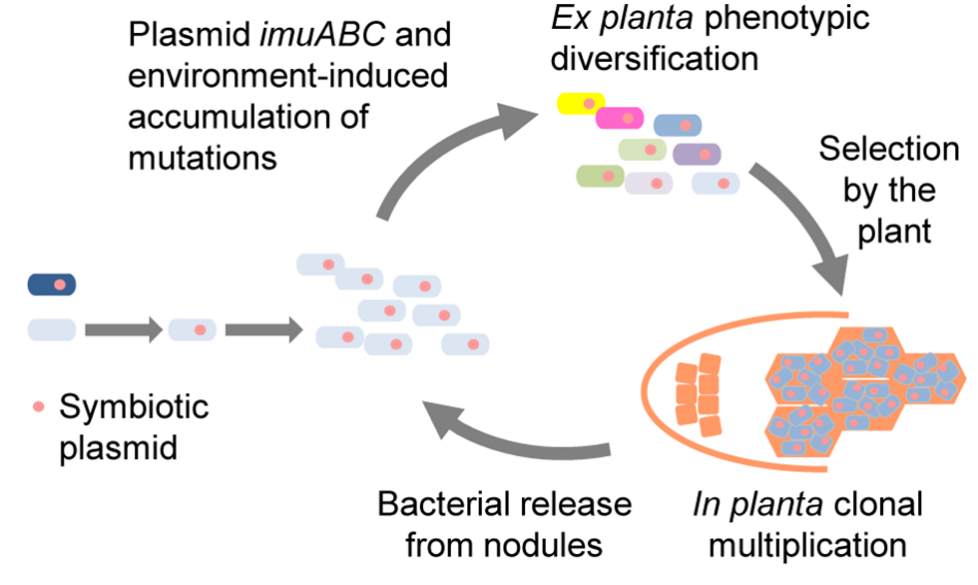
Model for symbiotic and mutagenic plasmid-driven evolution of rhizobia. Following horizontal transfer of a symbiotic plasmid to a soil bacterium, the recipient genome accumulates environment-induced mutations that lead to phenotypic diversification. The most beneficial variants are selected by the plant and clonally multiply within nodules before being released. Rounds of *ex planta* phenotypic diversification/plant selection/clonal multiplication may have driven the adaptation process *in natura*.

Competition experiments revealed that, after only five cycles of *ex planta*-*in planta* evolution *imuA2B2C2* ^+^ populations colonized *Mimosa* nodules much better than Δ*imuA2B2C2* populations, while *imuA2B2C2* ^+^ and Δ*imuA2B2C2* ancestors were found equally fit *in planta* (Figures 3B and S5B). This increase in fitness could reflect an improvement of the pre-symbiotic and symbiotic performances such as root colonization/attachment, root entry, nodule formation, and *in planta* multiplication and persistence. Alternatively it could reflect a better survival in the plant culture medium. Loss of the cassette in the chimeric *Ralstonia* indeed reduced its survival in Jensen with *Mimosa* (Figure S2B). Two lines of evidence however led us to exclude that cycles of plant-bacteria co-culture have selected for bacterial variants that had increased chances of being able to initiate a nodule exclusively because they better survived *ex planta*. First, the symbiotic advantage of *imuA2B2C2* ^+^ bacteria was more important in populations evolved *ex planta-in planta* than those evolved exclusively *ex planta* (Figures 3B, S5B, and S5C). Second, *ex planta-in planta* passages did not improve the survival of *imuA2B2C2* ^+^ populations in the Jensen medium with *Mimosa* (Figures 3C and S5D). Our results indicate that beneficial variants were enriched by *in planta* passages, likely through the selection by the plant of the most beneficial rhizospheric variants possibly at several stages. Altogether we propose that rounds of *ex planta imuABC*-mediated phenotypic diversification, selection by the plant of beneficial variants, and *in planta* clonal expansion of the selected variants accelerate symbiotic evolution (Figure 4).

### *imuABC* Cassettes Are Over-represented in pSym-rhizobia

**Figure 5:**
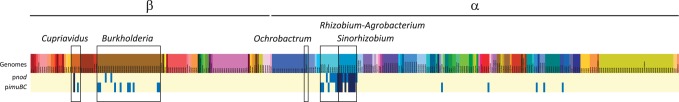
Distribution of plasmid *nodABC* (p*nod*) and plasmid *imuBC* (p*imuBC*) genes among α- and β-proteobacteria. Blue and yellow rectangles indicate presence and absence of genes in the corresponding genome, respectively, as assayed by BlastP analysis. Dark blue rectangles indicate *nodABC* and *imuBC* genes co-localized on the same plasmid. α- and β-proteobacteria are arranged according to their position on the core genome phylogeny. Species of the same genus are similarly colored. See Table S3 for details.

Rhizobia harboring nodulation and nitrogen fixation genes on plasmids (pSym-rhizobia) belong to four α-proteobacterial genera, *Agrobacterium*, *Rhizobium*, *Sinorhizobium*, and *Ochrobactrum*, and two β-proteobacterial genera, *Burkholderia* and *Cupriavidus*. Rhizobia emerged several times independently in these lineages by horizontal gene transfer, as attested by phylogenetic data and the observation that these genera contain both symbiotic and non-symbiotic species/strains [15]. To assess the overall contribution of *imu(A)BC* cassettes in the natural evolution of pSym-rhizobia we analyzed the 349 available genomes of α- and β-proteobacteria, representing 109 genera, for the presence of *imuBC* genes. More than half of the genomes possess a chromosomal *imuBC* genes but only 28 have a plasmid *imuBC* cassette (Table S3). We found that (i) 82% of all plasmid *imuBC* cassettes fall in the six genera that contain pSym-rhizobia, and (ii) 45% of the symbiotic plasmids carry a *imuBC* cassette (Figure 5; Table S3). Noteworthy, vestigial *imuBC* genes were found in some rhizobia (e.g., *Rhizobium* sp. IRBG74 GenBank, http://www.ncbi.nlm.nih.gov/Genbank, accession number CDI11787.1/CDI11788.1) suggesting that mutagenesis cassettes have been lost, possibly as a result of counter-selection of hypermutagenesis in well-adapted symbiotic populations. We thus speculate that symbiotic plasmid ImuBC error-prone DNA polymerases have enhanced the dissemination of symbiotic proficiency among α- and β-proteobacteria. Error-prone DNA polymerases may have been maintained in recipient genomes because they increase survival under stress conditions. In line with the proposal that *Cupriavidus* nodulation genes have been recently acquired from *Burkholderia* [20], the plasmid *imuB2C2* genes of *C. taiwanensis* are phylogenetically closer to *Burkholderia imuBC* cassettes than to the chromosomally encoded cassette (Figure S6). Interestingly *imuBC* genes are also found on large non-symbiotic plasmids, including the pAt and pTi virulence plasmids of the plant pathogen *Agrobacterium* (Figure S6), suggesting that *imuABC* cassettes also play(ed) a role in the spread of plasmid-encoded accessory biological functions.

### Conclusion

How complex phenotypic traits can be successfully transferred to evolutionary distant taxa is a poorly documented question despite its ecological and evolutionary importance. Whereas physical and genetic barriers restricting HGT have been identified [10], the many examples of successful HGT over large phylogenetic distances [13],[39] suggested the existence of environmental, genetic, or selective conditions favoring long-range HGT. Comparative genomics and experimental data have previously established the role of HGT in rhizobium evolution [18],[40],[41]. They also pointed out that activation and/or optimization of the symbiotic potential might rely on a combination of molecular events, involving integration of incoming symbiotic functions into pre-existing regulatory circuitries and recruitment, modulation or inactivation of local functions [15],[24], allowing the newly acquired functions to adjust to both the recipient cell and the new plant environment. Each plant indeed represents a complex ecosystem with specific requirements, e.g., in terms of immunity and metabolism, to which the bacterium must adapt.

Here, we describe a mechanism that may have facilitated post-HGT adaptation of emerging rhizobia to their new host *in natura* and better accounts for the great extant diversity of rhizobia (Figure 4). This may have facilitated the evolution of specific interactions with a wide range of legume species all over the Earth. Upon experimentally replaying rhizobium evolution, we provide conclusive evidence that the co-transfer of *imuABC* error-prone DNA polymerase genes with key symbiotic genes accelerated the overall evolution of a soil bacterium into a legume symbiont under plant selection pressure by transiently increasing genetic diversity and thus likely accelerating the exploration of the fitness landscapes. Evolution of microorganisms in response to changing environments relies on the natural selection of genetic variants harboring beneficial phenotypic traits [42]. Yet, standing genetic variation may not provide sufficiently adaptive variants when environmental changes are radical. It has previously been proposed that environment-induced increase in the rate of generation of genetic diversity could accelerate adaptive processes [43]–[45]. Although the molecular mechanisms underlying environment-induced mutagenesis are well known [45], its ultimate biological significance has been debated [46]. Our findings provide conclusive evidence for the role of environment-induced mutagenesis in the acquisition of a complex lifestyle trait.

HGT plays a ubiquitous role in the diversification of prokaryotes and exploration of new ecological niches [1],[2]. The presence of various types of error-prone polymerases on mobile genetic elements [34],[47], metabolic [48], and virulence plasmids of plant (Figure S6) and animal pathogens [49] suggests that co-transfer of environment-induced mutagenesis determinants with genes encoding complex phenotypic traits enhances the success of HGT, hence facilitating drastic lifestyle shifts.

So far, experimental evolution, combined in recent years with whole genome sequencing, has been predominantly used to study genetic adaptation to simple and well controlled conditions [29]. Our results highlight the potential of this approach to get further insight into very complex biological processes such as the emergence of symbiotic or pathogenic associations with multicellular eukaryotes.

## Materials and Methods

### Strains and Culture Conditions

Bacterial strains and plasmids used in this work are listed in Table S5. *Ralstonia* strains were routinely grown at 28°C on rich BG medium [24] or on MM minimal medium [24] supplemented with 2% glycerol. Antibiotics were used at the following concentrations (in micrograms per milliliter): nalidixic acid, 30; trimethoprim, 100; gentamycin, 25; kanamycin, 50; tetracycline, 10; spectinomycin, 40. *C. taiwanensis* strains were grown at 28°C on TY medium [50] supplemented with 6 mM CaCl_2_.

During evolution of A, B, C, G, H, and I lines the mean time bacteria spent in the plant tube before entering roots was estimated using nodulation kinetics of all ancestral and final clones. We calculated the mean time when half of the nodules appeared from which we subtracted 3 days (estimated time between the moment bacteria enter and the moment the nodule is visible).

For the SEC&R assay, 10^7^ bacteria grown overnight in rich BG medium were inoculated to a Gibson tube filled with quarter-strength Jensen medium alone (Jensen medium [51]) or containing in addition two *M. pudica* seedlings from surface-sterilized seeds as previously described [24]. Gibson tubes were incubated for 21 days—evaluated time bacteria spent in the plant medium in the selection cycle—at 28°C and under a 16-hour daylight period. For nodule-isolated clones the number of bacterial generations following plant entry was calculated using the formula “logn (number of bacteria per nodule)/logn2+3 logn(10^9^)/logn2,” taking into account free-living cultures before genomic DNA preparation.

For plasmid-loss experiments [52], bacterial strains in which the pLAFR6 plasmid had been previously introduced were grown for several days in different conditions and plasmid loss was determined by plating dilutions of bacterial cultures on agar medium with or without tetracycline.

For the 5-cycle evolution experiment of the *imuA2B2C2* ^+^ and Δ*imuA2B2C2* strains, ancestors were antibiotic resistant derivatives of the nodulating chimera GMI1000pRalta *hrpG* and its Δ*imuA2B2C2* mutant. GMI1000pRalta *hrpG* was chosen as founder since inactivation of this single regulatory gene allows nodulation in the first nodulating clones CBM212 and CBM349 [24]. The hypermutable and non-hypermutable phenotypes of the *imuA2B2C2* ^+^ and Δ*imuA2B2C2* ancestors were checked using the NalR assay after incubation 7 days in Jensen medium (Figure S5A).

The *imuA2B2C2* ^+^ and Δ*imuA2B2C2* strains were evolved using either serial *ex planta-in planta* or *ex planta* cycles. 10^8^ bacteria of ancestral strains (RCM861, RCM863, RCM865, and RCM1035) grown overnight in rich BG medium were inoculated to four or five Gibson tubes filled with quarter-strength Jensen medium containing two *M. pudica* plantlets. For *ex planta*-*in planta* cycles, all nodules were collected from each tube separately 21 days after inoculation, surface sterilized, and crushed. Nodule bacterial populations recovered from each tube were grown 24 hours at 28°C in BG medium and 10^8^ bacteria were used to inoculate a new tube containing two *M. pudica* plantlets in Jensen medium for the next cycle. On average, 18±6 nodules were collected per tube at each cycle. For *ex planta* cycles, the Jensen liquid medium of each plant tube was separately collected 7 days after inoculation and centrifuged 15 min at 5,000 rpm. Bacterial pellets were resuspended in 5 ml of BG medium and grown 24 hours at 28°C. 10^8^ bacteria from each culture were used to inoculate a new tube of plants for the next cycle. One tube per lineage was inoculated in each cycle. In each cycle samples of populations were stored at −80°C for further analysis. Non contamination between *imuA2B2C2* ^+^ and Δ*imuA2B2C2* populations was checked by PCR.

For fitness comparisons, different pairs of *imuA2B2C2* ^+^/Δ*imuA2B2C2* final populations were spread on plates from −80°C, resuspended in water and co-inoculated to *M. pudica* at a 1∶1 ratio (10^6^/10^6^ bacteria per tube of plants). Relative *in planta* fitness was evaluated by counting bacteria recovered from all nodules of ten plantlets 21 days after inoculation as previously described [25]. Relative *ex planta* fitness was evaluated by counting alive bacteria in Jensen medium 7 days after plant inoculation. Each competition was performed at least three times independently.

For survival measurements, single colonies of GMI1000pRaltaΔ*imuA2B2C2* (RCM567) and spectinomycin-derivatives of GMI1000pRalta (RCM1069) were grown overnight in rich BG medium and 10^7^ bacteria were inoculated to Gibson tubes filled with quarter-strength Jensen medium containing two *M. pudica* plantlets. Bacteria were counted by plating. Twelve independent experiments were performed.

### Sampling, Library, and DNA Preparation

In SEC&R experiments, *ex planta* and nodule populations were isolated as follows: nodules were collected, surface-sterilized as previously described [25], and ground separately in 1 ml of sterile water. The Jensen liquid medium was stirred to resuspend deposited matter, centrifuged, and the resulting pellet suspended in 1 ml of sterile water. Bacteria were plated on BG supplemented with the appropriate antibiotic and 19 to 20 clones were randomly chosen. Genomic DNA from each clone was prepared from a 1 ml overnight culture, using the Wizard Genomic DNA kit (Promega) according to the manufacturer's instructions. DNA concentrations were quantified using the Quant-iT PicoGreen dsDNA Assay kit (Invitrogen). DNA pools were constituted by mixing equimolar amounts of 19 or 20 genomic DNAs.

### Genome Re-sequencing

Individual clones and pools of clones were re-sequenced using the Illumina/Solexa technology, either the GA2X or HiSeq technology (Table S1). Sequence data production was performed by the C.E.A/IG/Genoscope (clones A16, B16, C16, G16, H16, I16, M16, N16, S16, and pools N1–3, J1, JM1–2), DNA Vision (pool JM6), or the PlaGe platform (other pools of clones and RCM252–RCM271 clones). Average sequence coverage of pools and clones from pools is indicated in Table S1.

High throughput sequencing (HTS) data were analyzed using the PALOMA bioinformatic pipeline implemented in the Microscope platform [53]. The current pipeline is a “Master” shell script that launches the various modules of the analysis (i.e., a collection of C homemade software) and controls for all tasks having been completed without errors. In a first step, the HTS data were preprocessed to assess its quality. This step includes options such as read trimming, merging, or splitting paired-end reads. In a second step, reads were mapped onto the reference replicons (RefSeq accession number NC_003295.fna and NC_003296.fna for the *R. solanacearum* str. GMI1000 chromosome and megaplasmid, respectively, RefSeq accession number NC_010528.fna, NC_010530.fna, and NC_010529.fna for the *C. taiwanensis* str. LMG19424 Chromosome 1, Chromosome 2, and pRalta, respectively) using the SSAHA2 package [54]. Only unique matches having an alignment score equal to at least half of their length were retained as seeds for full Smith-Waterman realignment [55] with a both sides five nucleotides extended region of the reference genome. All computed alignments were then screened for discrepancies between read and reference sequences and *in fine*, a score based on coverage, allele frequency, quality of bases, and strand bias was computed for each detected event to assess its relevance. The complete collections of events generated for all the clones from this study are available on the Microscope platform (https://www.genoscope.cns.fr/agc/microscope/expdata/evoProject.php).

To filter sequencing errors or undetected events in ancestors, the following criteria were applied: minimum reads on a position was 10 and strand ratio was 0.25. For clones, SNPs/indels having a score (high quality reads on the position/total reads on the position) <0.4 and an allele ratio (mutated reads on the position/total reads on the position) <0.61 were removed as well as SNPs/indels present in >30% of all clones. For pools of clones, SNPs/indels having a score <0.1 were removed as well as SNPs/indels present in >20% of pools. Point mutations already present in appropriate founder strains were not considered. Finally, mutations in genes Rsp0540, Rsp0641, Rsp0642, Rsp1180, Rsp1620, and Rsc0104 that harbor low complexity regions were removed. For pools of clones derived from *C. taiwanensis* LMG19424, which was not sequenced using the Illumina technology, mutations with an allele ratio >0.5 were considered as ancestral and removed. Filtered pool mutations are listed in Table S4.

For each mutation detected in pools of clones, the number of clones bearing each mutation was directly scored (pool JM3), or estimated using allele ratios (other pools).

### Mutation Analysis

For the validation of selected SNPs/indels, a ∼400 bp fragment containing the mutation was amplified by PCR either on individual clones or on pools of five or ten clones and subsequently sequenced by standard Sanger procedure. 97% of the 129 mutations tested in clones CBM124GenR, CBM212, CBM349, CBM356, A16, C16, G16, H16, M16, and N16 were validated by Sanger sequencing. Validations of mutations in pools or in clones RCM252–272 are indicated in Tables S1 and S4.

To calculate the *in planta* mutation rate, we used pools of CBM349 clones isolated from nodules. Considering that a single bacterium enters and multiplies within a nodule [56] to ∼10^6^ bacteria/nodule, we kept point-mutations having an allele ratio <0.9, i.e., likely having been acquired after root entry. From these mutations acquired in ∼110 generations (∼20 generations *in planta* and ∼90 generations for clone purification on plates), we calculated an estimated synonymous point-mutation rate (synonymous mutations/synonymous positions/110) and the CI was computed according to a binomial distribution using the R “stats” package and the *binom.test* function (http://www.R-project.org) [57].

To evaluate the evolutionary processes acting on newly arising mutations, we counted the number of synonymous, non-synonymous, and intergenic mutations in the sequenced regions. We used synonymous mutations of 38 GMI1000pRalta clones to build the mutation spectrum of the genome as they are expected to be the least affected by selection. For example, having n synonymous positions with G in the reference genome and x substitutions G→A detected in evolved genomes, the frequency of G→A changes is given by x/n. This assumes no multiple mutations in the same site. This assumption is consistent with the low density of mutations (1/13,130 bp) and the lack of identical mutations in different lineages. We simulated genome evolution using this mutation spectrum to obtain the expected number of synonymous, non-synonymous, and intergenic mutations. We did 1,000 such random experiments, which allowed drawing the distribution of the expected number of each mutation and thus placing intervals of confidence around the average values observed in the simulation. The dN/dS and dI/dS simulated values were then compared to the values observed in the experiments. The simulations were done separately for the chromosomes and for the pRalta plasmid because they have different GC compositions (respectively, ∼67% and 60%). Since replicons were not re-sequenced to completion, the analysis of each experiment was done independently to properly account for the fraction of the replicon covered by sequencing in that experiment.

### Fluctuation Tests

For each strain, an overnight culture in rich medium (BG) grown from a single colony was used to inoculate fresh BG medium that was subsequently divided into 23 individual cultures of ∼10^4^ bacteria/ml. The cultures were then grown to saturation (2 days at 28°C, on a rotary shaker) and an aliquot from each culture was plated on BG agar plates supplemented or not with nalidixic acid. Mutation rates were calculated using the Ma-Sandri-Sarkar maximum likelihood (MSS-ML) method [27], as implemented by the Falcor web tool [58].

### Mutagenesis Assays

For the NalR assay, strains were grown overnight in BG medium until early stationary phase. Quarter-strength Jensen filled Gibson tubes containing two *M. pudica* plantlets or not were inoculated with 4 ml of bacterial suspension adjusted to ∼2.5 10^9^ cfu/ml and incubated at 28°C. CBM124 was used as GMI1000pRalta strain. Total bacteria and nalidixic acid resistant clones were numerated by plating appropriate dilutions on selective media.

For CBM124/CBM124NalR competition experiments, ten spontaneous NalR derivatives of CBM124 were recovered by plating on BG supplemented with nalidixic acid an overnight culture grown in BG medium. Ten Gibson tubes filled with quarter-strength Jensen medium were each inoculated with a 1∶1 mixture of ten CBM124/CBM124NalR pairs. Bacteria were incubated for 7 days and numerated by plating on appropriate medium. Statistical significance of these results was determined using the unpaired two-tailed Student's test.

### Genetic Manipulations

Primers used for DNA amplification are listed in Table S6.

Spectinomycin-resistant derivatives of evolved clones and ancestors were constructed as previously described [25]. To construct the spectinomycin- or kanamycin-resistant derivative of GMI1000pRalta (RCM1069) and GMI1000pRalta *hrpG* (RCM865 and RCM1035), the *glmS*-RSc0179 intergenic region was amplified using the oCBM1574/oCBM1575 primer pair and cloned into the pGEM-T plasmid. *Sma*I-digested resistance gene cassettes ΩSpe from pHP45-Ω or ΩKan from pHP45-ΩKan were inserted into the *Sma*I site of the cloned region. The resulting plasmids were linearized with *Sca*I and introduced into the chimeric *Ralstonia* CBM124 and CBM1627 by natural transformation [24].

For the generation of pVO155 insertion mutants, internal regions of *imuB2* (pRalta_0100), *imuC2* (pRalta_0099), and *lexA* (RSc1304) were amplified by PCR using the oCBM1798/11799, oCBM1800/1801, and oCBM1808/1809 primer pairs, respectively, and cloned into pVO155 as *Bam*HI/*Xba*I restriction fragments. pVO155 derivatives were introduced into *C. taiwanensis*, *R. solanacearum*, and chimeric *Ralstonia* strains by triparental mating using the helper plasmid pRK600 in *E. coli* HB101. Transconjugants were selected on appropriate selective media and insertions of pVO155 in the appropriate genes were checked by PCR.

To generate the *imuA2B2C2* deletion mutants (RCM567, RCM861, and RCM863), flanking regions of this locus were separately amplified using the oCBM1756/1757 and oCBM1758/1759 primer pairs and subsequently cloned side-by-side into pGEM-T. *Sma*I-digested resistance gene cassettes ΩSpe from pHP45-Ω or ΩKan from pHP45-ΩKan were cloned between the two regions. The resulting plasmids were linearized with *Psi*I and introduced into chimeric *Ralstonia* by natural transformation [24]. Recombinant strains were selected on media with adequate antibiotics and gene exchanges were checked by PCR.

For complementation studies, the whole *imuA2B2C2* locus together with 500 bp upstream from *imuA2* was amplified using the oCBM1900/1901 primer pair and cloned as a *Avr*II/*Xba*I restriction fragment into pRCK-*lacZ1* plasmid [59]. The resulting plasmid was linearized with *Psi*I and the *imuA2B2C2* cassette integrated at the *glmS*-RSc0179 intergenic region by natural transformation in *Ralstonia* strains.

### Quantitative Reverse Transcription-PCR

To measure the expression of error-prone polymerases in Jensen medium, bacteria were first grown in rich BG medium to mid-exponential phase (OD600 = 0.6), then 10^10^ bacteria were centrifuged, resuspended in 1 ml of water, and transferred to 39 ml of Jensen medium at 28°C for 4 hours without agitation. 3 ml of cultures were then mixed with 2 vol of RNAprotect Bacteria Reagent (Qiagen), centrifuged for 10 min at 5,000 rpm, and stored at −80°C until RNA extraction. Bacteria were lysed in 200 µl of TE buffer containing 10 mg/ml lysozyme for 5 min at room temperature and RNAs were extracted using the RNeasy mini kit (Qiagen) according to manufacturer's instructions. RNA integrity was verified on a Bioanalyzer (Agilent). Reverse transcription was performed using Superscript II reverse transcriptase (Invitrogen) and random hexamers as primers on 1 µg of RNAs previously treated with TURBO DNase (Ambion). Real-time PCRs were run on a LightCycler system (Roche) using the FastStart DNA MasterPLUS SYBRGreen 1 kit (Roche) according to manufacturer's instructions. Oligonucleotide sequences used for quantitative PCR are listed in Table S6.

### Phylogeny and Distribution of Plasmid Cassettes

Genomes of α- and β-proteobacteria larger than 1 Mb were downloaded from GenBank RefSeq as available in February 2013. ImuABC cassettes were identified in protein coding sequences as genomically contiguous matches of PFAM profiles for Y- and C-polymerases (respectively, PF00817.15 and PF07733.7). Rhizobial genomes were identified by the presence of the common *nodABC* genes, respectively, PFAM domains PF02474, PF01522, PF00535. Profiles were searched using hmmer with eval <10^−5^ and only hits whose alignments covered more than half of the profile were selected. *nodABC* and *imuBC* genes were classified according to the replicon where they were encoded as chromosomal or plasmid-encoded.

Amino acid sequences of *imuBC* genes extracted from GenBank RefSeq complete genomes as available in February 2013 were aligned with Muscle [60] or MAFFT [61] and informative positions were extracted using BMGE [62]. Phylogenetic trees of individual genes and concatenates were constructed with PhyML [63] using optimal parameters given by Protest (LG matrix, 4-categories-discretized Gamma distribution for rate variation among sites, empirical frequencies of amino-acids) [64]. Node support values were computed by non-parametric bootstrap (1,000 experiments).

### Sequence Data

Sequence data for clones (SYMPA) or pools of clones (MUTA) are available on https://www.genoscope.cns.fr/agc/microscope/expdata/evoProject.php

## Supporting Information

Figure S1 **Mutation rates of** ***R. solanacearum*** **GMI1000, the GMI1000** ***mutS:aacC3-IV*** **mutant, the chimeric** ***Ralstonia*** **ancestor GMI1000pRalta (CBM124GenR), and the nodulating chimera CBM212, CBM349, and CBM356.** Frequency of nalidixic acid resistance was assessed by fluctuation tests. Error bars represent 95% CIs. Raw data are provided in Data S4.
(EPS)

Figure S2 **Survival and replication in Jensen and** ***Jensen-Mimosa*** **.** (A) and (B), GMI1000pRalta (red curves) and GMI1000pRaltaΔ*imuA2B2C2* (green curves) were individually incubated in Jensen-filled Gibson tubes containing (B) or not. (A) *M. pudica* plants and alive population sizes were estimated at different times. Twelve independent experiments were performed for each strain. GMI1000pRalta was labeled with the same spectinomycin-resistance cassette as GMI1000pRaltaΔ*imuA2B2C2* to avoid cassette-dependent growth modification (see Methods). The loss of the cassette significantly reduced the survival of the bacterium in the medium with *Mimosa* (*p*<0.001, t-test; *p*<0.001, Signed-rank test). (C,D, and E) Bacteria containing the replicative but unstable plasmid pLAFR6 were grown in log phase for several days in rich BG medium (C), in minimal MM 2% glycerol medium (D), or inoculated into Jensen-filled Gibson tubes containing or not *M. pudica* plantlets (E). The presence of pLAFR6 was determined by plating bacteria on appropriate medium. Dots represent independent replicates from three independent experiments, diamonds represent the mean. The rate of plasmid loss per generation was similar (R^2^ = 0.9601) in (C) (*y* = −0.0297*x*+0.9748) and (D) (*y* = −0.0276*x*+1.0869) and thus independent from the culture medium. On the basis of this rate, we estimated a maximum of five and three generations, respectively after 21 days in Jensen or Jensen *Mimosa*, suggesting that cells expressing the growth advantage in stationary phase phenotype [65] were not significantly selected during this period. Raw data are provided in Data S5.
(EPS)

Figure S3 **The** ***imuA2B2C2*** **cassette.** (A) pRalta *imuA2*, *imuB2*, and *imuC2* genes are organized in one operon, as assessed by RT-PCR on *C. taiwanensis* RNAs, and preceded by a typical β/γ-proteobacterial LexA binding SOS box (CTGTN_8_ACAG) [34]. (B) In Jensen medium the expression of *imuA2*, *imuB2*, and *imuC2* in GMI1000pRalta depends on LexA, the negative regulator of the SOS response. Bacteria were grown in exponential phase in rich Phi medium prior to inoculation to Jensen medium and incubated for 4 hours. Gene expression was measured by qRT-PCR and normalized by three housekeeping genes *rplA*, *rpoA*, and *dnaA*. Values are averages ± standard deviations from three independent experiments. Raw data are provided in Data S6.
(EPS)

Figure S4 **Kinetics and genetics of NalR mutation frequency.** (A) The frequency of NalR mutants regularly increased with time for both *R. solanacearum* GMI1000 and the chimeric *Ralstonia* GMI1000pRalta after incubation in Jensen medium suggesting dependence on the severity of the stress. Values are from seven independent replicates. Asterisks indicate significant differences between GMI1000 and GMI1000pRalta (*p*-values from Wilcoxon test <0.01). (B) For each strain, the frequency of spontaneous NalR mutants in bacterial populations is plotted before (T0) and after (T7) a 7-day incubation in Jensen plus *Mimosa*. Values are from ten to 12 independent replicates. Asterisks indicate T7 values significantly different from GMI1000 (two-tailed Kruskal-Wallis test, *p*<0.05); T0 values are not significantly different (*p*>0.05). Horizontal bars represent medians. Raw data are provided in Data S7.
(EPS)

Figure S5 **Competitions between** ***imuA2B2C2*** **^+^ and Δ** ***imuA2B2C2*** **strains and populations.** (A) The *imuA2B2C2* ^+^ and Δ*imuA2B2C2* chimeric ancestors, from which populations were derived via *ex planta - in planta* or *ex planta* lineages, were respectively confirmed as being mutable and non mutable in Jensen using the NalR assay. SpeR, spectinomycin-resistant strain. KanR, kanamycin-resistant strain. Values are from eight to ten independent replicates. Asterisks indicate T7 values significantly different from Δ*imuA2B2C2* strains (multiple comparison after Kruskal-Wallis test, *p*<0.05). T0 values are not significantly different (*p*>0.05). Horizontal bars represent medians. (B) Percentage of *imuA2B2C2* ^+^ strains in nodule populations following co-inoculation with pairs of An-*imu* ^+^(i) and An-Δ*imu*(i) ancestors or with each pair of Ev-*imu* ^+^(i) and Ev-Δ*imu*(i) populations evolved through *ex planta-in planta* cycles (see Figure 3A). Nodule bacteria were counted 21 days after inoculation. Values are from three to five independent competition experiments. *Indicates significant differences between *imu* ^+^ and Δ*imu* ancestors/populations for each competition experiment. Horizontal bars represent means. (C) Percentage of *imuA2B2C2* ^+^ strains in nodule populations following co-inoculation with pairs of An-*imu^+^*(i) and An-Δ*imu*(i) ancestors or with each pair Ev-*imu* ^+^(i′) and Ev-Δ*imu*(i′) populations evolved through *ex planta* cycles (see Figure 3A). Nodule bacteria were counted 21 days after inoculation. Values are from three independent competition experiments. *Indicates significant differences between *imu* ^+^ and Δ*imu* ancestors/populations for each competition experiment. Horizontal bars represent means. (D) Percentage of *imuA2B2C2* ^+^ strains in Jensen medium with *Mimosa* following co-inoculation with pairs of An-*imu* ^+^(i) and An-Δ*imu*(i) ancestors or with each pair of Ev-*imu* ^+^(i) and Ev-Δ*imu*(i) populations evolved through *ex planta-in planta* cycles (see Figure 3A). Bacteria recovered from the Jensen medium were counted 7 days after inoculation of Gibson tubes containing *Mimosa* plants. Values are from three independent competition experiments. *Indicates significant differences between *imu* ^+^ and Δ*imu* ancestors/populations for each competition experiment. Horizontal bars represent means. Raw data are provided in Data S3.
(EPS)

Figure S6 **Phylogenetic tree of the ImuBC cassette.** Maximum-likelihood phylogenetic tree of the concatenated ImuBC amino-acid sequences. Sequences were chosen among completely sequenced strains, with a focus on α- and β-rhizobia. Numbers at the nodes indicate bootstraps (1,000 experiments). *Frankia* sp. Eu1c was used to root the tree. When appropriate, names of the plasmids carrying an ImuBC copy are indicated in brackets. Plasmid cassettes are in green and rhizobia are underscored. Asterisks indicate symbiotic plasmids. Clades containing rhizobia are shaded in grey. Note the position of the *C. taiwanensis* and *C. necator* plasmid cassettes that are more closely related to *Burkholderia* sp. cassettes than to *Cupriavidus* sp. chromosomal cassettes. Abbreviations: Ac., *Acidovorax*; Ag., *Agrobacterium*; Bo., *Bordetella*; Br., *Brucella*; Bu., *Burkholderia*; Ca., *Caulobacter*; Cu., *Cupriavidus*; Fr., *Frankia*; Me., *Mesorhizobium*; Mt., *Methylobacterium*; P., *Pseudomonas*; Ra., *Ralstonia*; Ro., *Rhodobacter*; Rh., *Rhizobium*; S., *Sinorhizobium*; V., *Vibrio*; X., *Xanthomonas*. Alignments are provided in Data S8.
(EPS)

Table S1 **Number of point mutations detected in pools of clones.** The clones were randomly isolated 21 days after inoculation of a founder strain in a Gibson tube filled with Jensen medium and containing or not *M. pudica* plantlets. Each pool contains equimolar concentration of DNA from 19 (J4) or 20 (others) clones from a same compartment. ^a^These three nodules were collected from the same plant individual. All other pools were independent experiments. ^b^In these pools the 20th clone was CBM212, which served as control. ^c^Tubes were inoculated with CBM124 (pool J5) or CBM63 (poolJM3), two clones obtained from independent transfer of pRalta to GMI1000, or with a GenR derivative of CBM124 (pools J1–J4, JM1, JM2). ^d^The 20 clones of this pool were individually sequenced (RCM252 to RCM271). ^e^Mutations were randomly chosen for Sanger validation. ^f^Number of different mutations. G, GA2X; H, HiSeq2000; na, non applicable. Details are provided in Table S4.
(XLSX)

Table S2 **Type of selection acting on evolved clones and pools of clones.** *Ratio of the number of non-synonymous substitutions per non-synonymous site to the number of synonymous substitutions per synonymous site. **Ratio of the number of intergenic substitutions per intergenic site to the number of synonymous substitutions per synonymous site.
(DOCX)

Table S3 **Distribution of** ***nodABC*** **and** ***imuBC*** **genes in α- and β-proteobacteria.** Y and N indicate presence and absence, respectively. Numbers in brackets indicate the number of chromosomic or plasmid *imuBC* cassettes. Genera that contain rhizobial species with plasmid *nodABC* genes are highlighted in grey. *Note that *Agrobacterium* and *Ochrobactrum* contain rhizobia [66],[67] yet no rhizobial strain has been sequenced within these genera. *Agrobacterium* and *Rhizobium* genera do not form two separate clades and have been proposed to be amalgamated [68]. #*imuBC* genes present on the symbiotic plasmid.
(XLSX)

Table S4 **Mutations detected in pools of clones.** Sheet 1: *Ralstonia* pools; sheet 2: *Ralstonia* clones of JM3 pools; sheet 3: *C. taiwanensis* pools. a, position on the replicon; b, position on the CDS. c, for intergenic mutations; d, mutations are validated by Sanger sequencing of PCR fragments from individual clones (A), sub-pools of five clones (B) or ten clones (C). Information is: nucleotide change/SNP, INSertion or DELetion/transition (ts) or transversion (tv)/codon change/aa change/nonsynonymous or synonymous mutation/score/allele ratio/sequencing technology/single end (se) or paired end (pe)/automatic.
(XLSX)

Table S5 **Strains and plasmids used in this study.** *Carries *tra* and *mob* genes [69].
(DOCX)

Table S6 **Primers used in this study.**
(DOCX)

Data S1 **Raw data for** **Figure 1** **.**
(XLSX)

Data S2 **Raw data for** **Figure 2** **.**
(XLSX)

Data S3 **Raw data for** **Figures 3** **and S5.**
(XLSX)

Data S4 **Raw data for Figure S1.**
(XLSX)

Data S5 **Raw data for Figure S2.**
(XLSX)

Data S6 **Raw data for Figure S3.**
(XLSX)

Data S7 **Raw data for Figure S4.**
(XLSX)

Data S8 **Alignment of** ***imuBC*** **genes (Figure S6).**
(TXT)

## Abbreviations

HGT: horizontal gene transfer
SEC&R assay: single evolution cycle and re-sequencing assay
