# Supplementary material for: Transient Hypermutagenesis Accelerates the Evolution of Legume Endosymbionts following Horizontal Gene Transfer
Source: PLoS Biol. 2014 Sep 2;12(9):e1001942. doi: 10.1371/journal.pbio.1001942 (PMC4151985; doi:10.1371/journal.pbio.1001942)
Supplement: Table S2 — Type of selection acting on evolved clones and pools of clones. *Ratio of the number of non-synonymous substitutions per non-synonymous site to the number of synonymous substitutions per synonymous site. **Ratio of the number of intergenic substitutions per intergenic site to the number of synonymous substitutions per synonymous site. (DOCX) [file pbio.1001942.s008.docx]

| **Table S2. Type of selection acting on evolved clones and pools of clones.** | | | | | |  |  |
| --- | --- | --- | --- | --- | --- | --- | --- |
|  |  | dN/dS* | | | dI/dS** | | |
| Conditions | Replicon | Observed | Expected | p-value | Observed | Expected | p-value |
| Final clones (A16, B16, C16, G16, H16, I16, M16, N16, S16) | Chromosome 1 | 0.431 | 0.720 | **0.001** | 0.586 | 0.784 | 0.218 |
|  | Chromosome 2 | 0.508 | 0.735 | **0.011** | 1.334 | 0.794 | **0.044** |
|  | pRalta | 0.669 | 0.617 | 0.305 | 0.660 | 0.710 | 0.464 |
| Jensen pools (J1-5) | Chromosome 1 | 0.317 | 0.792 | **0.004** | 0.934 | 0.868 | 0.413 |
|  | Chromosome 2 | 0.826 | 0.951 | 0.512 | 2.534 | 1.104 | 0.067 |
|  | pRalta | 0.424 | 0.653 | 0.198 | 0.845 | 0.765 | 0.358 |
| Jensen-Mimosa pools (JM1-3) | Chromosome 1 | 0.616 | 0.731 | 0.185 | 0.857 | 0.807 | 0.385 |
|  | Chromosome 2 | 0.509 | 0.746 | **0.031** | 1.408 | 0.842 | 0.091 |
|  | pRalta | 0.748 | 0.620 | 0.213 | 0.483 | 0.715 | 0.206 |
| All pools (J1-5, JM1-3) | Chromosome 1 | 0.524 | 0.720 | **0.020** | 0.878 | 0.793 | 0.348 |
|  | Chromosome 2 | 0.560 | 0.737 | 0.082 | 1.590 | 0.812 | **0.020** |
|  | pRalta | 0.614 | 0.613 | 0.430 | 0.626 | 0.709 | 0.392 |
| * ratio of the number of non-synonymous substitutions per non-synonymous site to the number of synonymous substitutions per synonymous site | | | | | | | |
| ** ratio of the number of intergenic substitutions per intergenic site to the number of synonymous substitutions per synonymous site | | | | | | | |
