# Supplementary material for: Transient Hypermutagenesis Accelerates the Evolution of Legume Endosymbionts following Horizontal Gene Transfer
Source: PLoS Biol. 2014 Sep 2;12(9):e1001942. doi: 10.1371/journal.pbio.1001942 (PMC4151985; doi:10.1371/journal.pbio.1001942)
Supplement: Table S5 — Strains and plasmids used in this study. *Carries tra and mob genes [69]. (DOCX) [file pbio.1001942.s011.docx]

**Table S5.** Strains and plasmids used in this study

| **Bacterium** | **Strain** | **Relevant characteristics** | **Reference/source** |
| --- | --- | --- | --- |
| *E. coli* | DH5α | *F recA lacZ*DM15 | Bethesda Research Laboratory |
| *R. solanacearum* | GMI1000 | wild-type strain (phylotype IA) isolated from tomato in French Guyana | [1] |
|  | GMIΔ*mutS* | *mutS::aac*C3-IV mutant derivative of GMI1000, GenR | [2] |
|  | RCM872 | GMI1000::*imuA2B2C2*, KanR | This study |
| Chimeric *Ralstonia* | CBM124 | GMI1000 pRalta::Tri, TriR | [3] |
|  | CBM124GenR | GMI1000 Rsp1236::pCZ367 pRalta::Tri, TriR, GenR | [3] |
|  | RCM552 | CBM124 (pLAFR6), TriR, TetR | This study |
|  | CBM63 | GMI1000 pRalta::Tn5-B13S, TetR | This study |
|  | CBM212 | spontaneous nodulating clone isolated from *M. pudica* inoculated with CBM124GenR, TriR, GenR | [3] |
|  | CBM349 | spontaneous nodulating clone isolated from *M. pudica* inoculated with CBM124GenR, TriR, GenR | [3] |
|  | CBM356 | spontaneous nodulating clone isolated from *M. pudica* inoculated with CBM124GenR, TriR, GenR | [3] |
|  | RCM567 | CBM124 ∆*imuA2B2C2*, TriR, SpeR | This study |
|  | RCM649 | CBM124 *imuC2*::pVO155, TriR KanR | This study |
|  | RCM873 | CBM124::*imuA2B2C2* ∆*imuA2B2C2*, TriR, SpeR, KanR | This study |
|  | RCM555 | CBM124 lexA::pVO155, TriR, KanR | This study |
|  | CBM1627 | CBM124 *hrpG,* TriR | [4] |
|  | RCM861 | CBM1627::ΩSpe, ∆*imuA2B2C2*, TriR, SpeR | This study |
|  | RCM863 | CBM1627::ΩKan, ∆*imuA2B2C2*, TriR, KanR | This study |
|  | RCM865 | CBM1627::ΩSpe, TriR, SpeR | This study |
|  | RCM1035 | CBM1627::ΩKan, TriR, KanR | This study |
|  | RCM1069 | CBM124::ΩSpe, TriR, SpeR | This study |
| *C.taiwanensis* | LMG19424 | Wild-type strain isolated from *Mimosa pudica* in Taiwan | [5] |
|  | CBM832 | LMG19424 derivative resistant to Str | [3] |
|  | CBM2440 | CBM832 *imuB2*::pVO155, StrR, KanR | This study |
| **Plasmids** | **Name** | **Relevant characteristics** | **Reference/source** |
|  | pRalta | likely auto-transferable* symbiotic plasmid of LMG19424, 0.5 Mb | [6] |
|  | pGEM-T | Cloning vector, AmpR | Promega |
|  | pVO155 | Derivative plasmid of pUC19 containing the promoterless gus *(uidA)* gene, KanR, AmpR | [7] |
|  | pLAFR6 | pLAFR1 with trp terminator, TcR | [8] |
|  | pRCK-lacZ1 | Plasmid for *R. solanacearum* chromosomal integration of genes at the *glmS* intergenic region, KanR | [9] |
|  | pHP45Ω | pBR322 derivative carrying a SpeR cassette, SpeR | [10] |
|  | pHP45Ω-Km | pBR322 derivative carrying a KanR cassette, KanR | [11] |

* carries *tra* and *mob* genes [12]

References

1. Boucher CA, Barberis PA, Trigalet AP, Demery DA (1985) Transposon mutagenesis of *Pseudomonas solanacearum*: isolation of Tn5-induced avirulent mutants. J Gen Microbiol 131: 2449-2457.

2. Mercier A, Bertolla F, Passelègue-Robe E, Simonet P (2007) Natural transformation-based foreign DNA acquisition in a *Ralstonia solanacearum mutS* mutant. Res Microbiol 158: 537-544.

3. Marchetti M, Capela D, Glew M, Cruveiller S, Chane-Woon-Ming B, et al. (2010) Experimental evolution of a plant pathogen into a legume symbiont. PLoS Biol 8.

4. Guan SH, Gris C, Cruveiller S, Pouzet C, Tasse L, et al. (2013) Experimental evolution of nodule intracellular infection in legume symbionts. ISME J 7: 1367-1377.

5. Chen WM, Laevens S, Lee TM, Coenye T, De Vos P, et al. (2001) *Ralstonia taiwanensis* sp nov., isolated from root nodules of *Mimosa* species and sputum of a cystic fibrosis patient. Int J Syst Evol Microbiol 51: 1729-1735.

6. Amadou C, Pascal G, Mangenot S, Glew M, Bontemps C, et al. (2008) Genome sequence of the beta-rhizobium *Cupriavidus taiwanensis* and comparative genomics of rhizobia. Genome Res 18: 1472-1483.

7. Oke V, Long SR (1999) Bacteroid formation in the Rhizobium-legume symbiosis. Curr Opin Microbiol 2: 641-646.

8. Huynh TV, Dahlbeck D, Staskawicz BJ (1989) Bacterial blight of soybean: regulation of a pathogen gene determining host cultivar specificity. Science 245: 1374-1377.

9. Monteiro F, Solé M, van Dijk I, Valls M (2012) A chromosomal insertion toolbox for promoter probing, mutant complementation, and pathogenicity studies in *Ralstonia solanacearum*. Mol Plant Microbe Interact 25: 557-568.

10. Prentki P, Krisch HM (1984) *In vitro* insertional mutagenesis with a selectable DNA fragment. Gene 29:303-313.

11. Fellay R, Frey L, Krish HM (1987) Interposon mutagenesis of soil and water bacteria: a family of DNA fragments designed for *in vitro* insertional mutagenesis of gram negative bacteria. Gene 52:147-154.

12. Guglielmini J, Quintais L, Garcillan-Barcia MP, de la Cruz F, Rocha EPC (2011) The repertoire of ICE in prokaryotes underscores the unity, diversity, and ubiquity of conjugation. PLoS Genet 7.
