# Supplementary material for: Transient Hypermutagenesis Accelerates the Evolution of Legume Endosymbionts following Horizontal Gene Transfer
Source: PLoS Biol. 2014 Sep 2;12(9):e1001942. doi: 10.1371/journal.pbio.1001942 (PMC4151985; doi:10.1371/journal.pbio.1001942)
Supplement: Table S6 — Primers used in this study. (DOCX) [file pbio.1001942.s012.docx]

**Table S6.** Primers used in this study.

| Primer name | Primer sequence 5'-3' | Use |
| --- | --- | --- |
| oCBM1574 | CGTGGTCGGAAGAATTCAC | amplify the *glmS*-RSc0179 intergenic region |
| oCBM1575 | TGGAGACCGTCATGTACCAA | amplify the *glmS*-RSc0179 intergenic region |
| oCBM1756 | TATCATCGATGAAGGCGAGCATCTCGGACC | amplify upstream region of *imuA2B2C2* |
| oCBM1757 | TCTACCCGGGAAAGTGACGGATGGATGGTT | amplify upstream region of *imuA2B2C2* |
| oCBM1758 | TCTACCCGGGATCTATGGTCAGTGGCAATG | amplify downstream region of *imuA2B2C2* |
| oCBM1759 | GATCGAATTCGCCGAACCAACATTGTCACC | amplify downstream region of *imuA2B2C2* |
| oCBM1798 | TATCGGATCCATTCACGCCTTGCCCGC | amplify fragment of *imuB2* to clone into pVO155 |
| oCBM1799 | TATCTCTAGACAATGCCACATGGAACCGCT | amplify fragment of *imuB2* to clone into pVO155 |
| oCBM1800 | TATCGGATCCATCTGCTACTGCCTGGGCAT | amplify fragment of *imuC2* to clone into pVO155 |
| oCBM1801 | TATCTCTAGACATCGTTGGGACAACTCGGC | amplify fragment of *imuC2* to clone into pVO155 |
| oCBM1808 | TATCGGATCCTTCTCGTCGCCGAATGCGG | amplify fragment of *lexA* to clone into pVO155 |
| oCBM1809 | TATCTCTAGAAGGATGCCGGCATCGCGCAT | amplify fragment of *lexA* to clone into pVO155 |
| oCBM1900 | TCTAGAGGGTACTGCGTTGTC | amplify the *imuA2B2C2* cassette |
| oCBM1901 | CCTAGGCGTGGCTAATCAGGTTTC | amplify the *imuA2B2C2* cassette |
| oCBM1868 | TCTGAGCCTGGTCAAGGAAT | amplify a *rplA* fragment for qPCR |
| oCBM1869 | CTTCGCATCGATACCCAGTT | amplify a *rplA* fragment for qPCR |
| oCBM1870 | TATATTGGCGACCTGATCCA | amplify a *rpoA* fragment for qPCR |
| oCBM1871 | CGAGGACTTCCTTGATCTCG | amplify a *rpoA* fragment for qPCR |
| oCBM1872 | AAGACCCACTTGATCCATGC | amplify a *dnaA* fragment for qPCR |
| oCBM1873 | GCCTTCACTACGTCGGAAAC | amplify a *dnaA* fragment for qPCR |
| oCBM1874 | GCCTTGATTCTCTGGCAGTC | amplify a *imuA2* fragment for qPCR |
| oCBM1875 | ACCACGAAGAAGAGCGTGTC | amplify a *imuA2* fragment for qPCR |
| oCBM1876 | AGGACCACACCTGCTACTGG | amplify a *imuB2* fragment for qPCR |
| oCBM1877 | CCGAAGAGACCATGGAGGTA | amplify a *imuB2* fragment for qPCR |
| oCBM1878 | GCCAATCTCTGCCAATTCTC | amplify a *imuC2* fragment for qPCR |
| oCBM1879 | CACCTCGTCAGGGTATTCGT | amplify a *imuC2* fragment for qPCR |
